# Silencing of R-Spondin1 increases radiosensitivity of glioma cells

**DOI:** 10.18632/oncotarget.3395

**Published:** 2015-03-12

**Authors:** Xuefeng Gu, Xuefeng Wang, Hong Xiao, Guoda Ma, Lili Cui, You Li, Haihong Zhou, Wandong Liang, Bin Zhao, Keshen Li

**Affiliations:** ^1^ Institute of Neurology, Guangdong Medical College, Zhanjiang 524001, China; ^2^ Guangdong Key Laboratory of Age-Related Cardiac and Cerebral Diseases, Affiliated Hospital of Guangdong Medical College, Zhanjiang 524001, China; ^3^ Department of Neurosurgery, Fourth Affiliated Hospital of Harbin Medical University, Harbin 150001, China; ^4^ Department of Neurology, Affiliated Hospital of Guangdong Medical College, Zhanjiang 524001, China; ^5^ Renji College, Wenzhou Medical University, Wenzhou 325000, China

**Keywords:** Rspo1, glioma, radiation

## Abstract

Although radiation therapy is the most effective postoperative adjuvant treatment, it does not substantially improve the long-term outcomes of glioma patients because of the characteristic radioresistance of glioma. We found that R-Spondin1 (Rspo1) expression was elevated in high-grade gliomas and was associated with worse overall survival and disease-free survival. Rspo1 expression was also associated with reduced survival rates in glioma patients after treatment with radiotherapy and temozolomide (RT-TMZ). Importantly, Rspo1 was dramatically upregulated after radiation treatment in patients with glioma. Rspo1 silencing by shRNA potentiated glioma cell death upon radiation treatment. In a xenograft nude mouse model, combining radiation and silencing of Rspo1 potentiated tumor growth inhibition. Thus, combining radiotherapy with silencing of Rspo1 is a potential therapeutic approach.

## INTRODUCTION

The R-Spondin (roof plate-specific spondin) family is a group of four secreted proteins (Rspo1–4) known to be potent agonists of Wnt signaling with important functions in development, stem cell survival and differentiation, and oncogenesis [1]. All four Rspo family members contain an N-terminal signal peptide and a C-terminal region rich with positively charged amino acids [2]. Rspo1, a secreted ~35 kDa molecule, binds to leucine-rich repeat-containing G protein-coupled receptor (LGR) 4–6 and synergizes with soluble Wnt3a to induce LRP6 phosphorylation and promote cytoplasmic stabilization as well as nuclear accumulation of β-catenin for cellular proliferation, differentiation and stem cell maintenance [3]. Transgenic expression of *Rspo1* induces significant enlargement of the small and large intestines [4], whereas administration of recombinant or adenoviral Rspo1 alleviates intestinal injury and oral mucositis induced by chemoradiotherapy [5–7]. Based upon the findings that Rspo1 is protective against radiation-induced gastrointestinal syndrome, we hypothesize that Rspo1 may be involved in the radioresistance of tumor cells to radiation therapy.

Gliomas are the most common primary tumors arising in the brain. Glioblastomas are high-grade gliomas that are among the most aggressive and difficult-to-treat human cancers [8]. Despite the use of conventional therapeutic modalities, such as surgery, chemotherapy, and radiotherapy, the prognosis of patients remains poor. Radiation therapy is a core therapy for malignant glioma, which consists of concomitant chemoradiotherapy with temozolomide after debulking surgery [9]. However, resistance to radiation occurs in most patients, and the underlying molecular mechanisms of radioresistance are not fully understood. New therapeutic strategies must be developed for improved long-term management of these tumors. Enhancing the effects of radiation, the primary adjuvant treatment for glioma, may increase the survival and quality of life of patients.

In this study, we observed that the expression of Rspo1 was significantly associated with poor overall survival and reduced survival of patients with gliomas after treatment with radiotherapy and temozolomide (RT-TMZ). In particular, we showed that radiation treatment triggered significant upregulation of Rspo1 in patients with gliomas, and increased cell death was observed upon silencing of Rspo1 via shRNA. As a result, we showed that the combination of radiotherapy with Rspo1 silencing potentiated tumor growth inhibition in a xenograft nude mouse model.

## RESULTS

### Overexpression of Rspo1 in human glioma tissues and glioma cell lines

**Figure 1:**
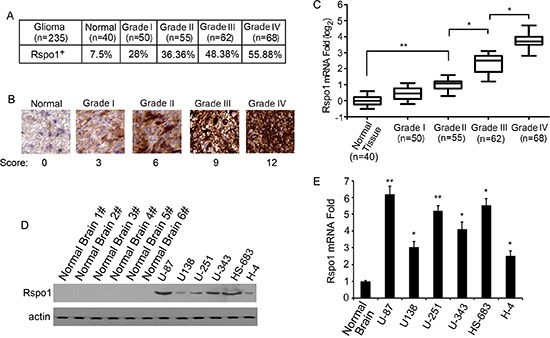
Increased expression of Rspo1 in gliomas **(A)** Rspo1 expression is presented as a percentage in normal brain tissues and Grade I-IV gliomas. Positive or negative expression of Rspo1 was defined as described in Materials and Methods according to the immunostaining score. **(B)** Levels of Rspo1 in normal tissues compared with Grade I-IV glioma tissues determined by immunostaining with the anti-Rspo1 antibody. Normal brain tissues were obtained from automobile accident victims without gliomas. Scale bar: 50 μm. **(C)** The expression of Rspo1 was verified by qRT-PCR in normal tissues and glioma samples. The top and bottom of each box plot represent the 5th and 95th percentiles, respectively, the middle line represents the mean value and the whiskers represent one standard deviation higher and lower than the mean. **(D)** Expression of Rspo1 in human glioma cell lines was determined by immunoblotting using an antibody against Rspo1; actin was used as a loading control. **(E)** Levels of Rspo1 mRNA in normal brain tissues and glioma cell lines determined by qRT-PCR analysis. Error bars: ± S.D. *p < 0.05, **p < 0.01. The results represent at least three separate experiments.

Immunohistochemical analysis was performed to determine the specific expression of Rspo1 protein in human gliomas. Using an antibody against Rspo1 for immunostaining, we examined tissue samples from 235 patients with a pathological diagnosis of astrocytic glioma. Immunoreactivity for the Rspo1 antigens was observed in 28% (14/50) of the patients with WHO Grade I glioma, 36.36% (20/55) of the patients with WHO Grade II glioma, 48.38% (30/62) of the patients with WHO Grade III glioma, 55.88% (38/68) of the patients with WHO Grade IV glioma and 7.5% (3/40) of normal brain tissues from automobile accident victims without glioma (Fig. 1A). Notably, the Rspo1 immunostaining was much stronger in high-grade gliomas than in low-grade gliomas (Fig. 1B). To confirm the upregulation of Rspo1, real-time qRT-PCR analysis was performed using normal brain tissue samples and glioma tissue samples. Consistent with the results of the immunohistochemical analysis, elevated levels of Rspo1 mRNA were detected in high-grade glioma tissues compared with low-grade gliomas and normal brain tissue samples (Fig. 1C). We next examined the expression of Rspo1 in glioma cell lines using Western blotting assays with anti-Rspo1 antibodies. Compared with normal brain tissue lysate, elevated Rspo1 expression was observed in all six glioma cell lines (Fig. 1D). These results were also confirmed by real-time qRT-PCR analysis (Fig. 1E).

### Expression of Rspo1 correlates with shortened survival and decreased survival rates after RT-TMZ therapies

**Figure 2:**
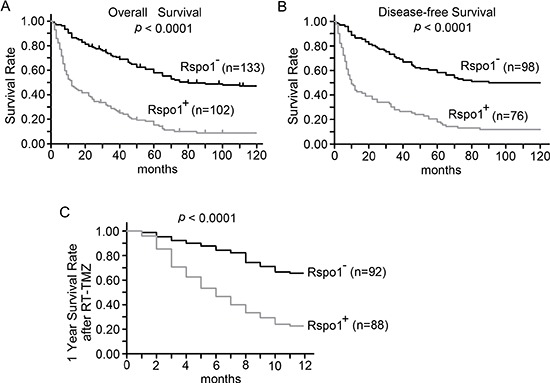
Expression of Rspo1 is associated with low survival in patients with glioma **(A, B)** Kaplan-Meier analyses were performed according to Rspo1 protein expression scores in glioma patients. Immunostaining scores of 0–2 and ≥ 3 were considered to indicate negative and positive expression of Rspo1, respectively. The overall survival of patients with positive Rspo1 expression was significantly shorter (A). The disease-free survival of patients with positive Rspo1 expression was also significantly shorter (B). **(C)** One-year survival rates of glioma patients after treatment with RT-TMZ based on their immunoreactivity scores. Scores of 0–2 and ≥ 3 were considered to indicate negative and positive expression of Rspo1, respectively. The total survival rates of Rspo1-positive patients and Rspo1-negative patients were compared using Kaplan-Meier analyses.

We also evaluated whether immunoreactivity against Rspo1 was correlated with overall survival in 235 patients with glioma. We observed that upregulation of Rspo1 predicted shorter overall survival and disease-free survival in patients with gliomas (Fig. 2A and 2B). Multivariate survival analysis using the Cox proportional hazards model further indicated that upregulation of Rspo1 was correlated with a higher hazard ratio (HR) and poor clinical outcomes (overall survival, *p* = 0.008, HR 7.778; for disease-free survival, *p* = 0.012, HR 6.357) (Table 1). We therefore investigated the role of Rspo1 in therapies using radiation concomitantly with adjuvant temozolomide (RT-TMZ). We observed that the one-year survival rates were 66.3% (61/92) for patients with negative Rspo1 expression and 23.86% (21/88) for patients with positive Rspo1 expression (*p* < 0.0001, Fig. 2C). These results highlight the clinical importance of Rspo1 in determining the prognosis of patients with gliomas and reveal a new target for glioma therapy.

**Table 1:** Multivariate Cox regression analysis of the expression of Rspo1 in glioma

Variable		Overall Survival		Disease-Free Survival	
		HR (95% CI)	*p* Value	HR (95% CI)	*p* Value
Rspo1 expression		7.778 (2.136–9.502)	**0.008**	6.357 (2.328–7.416)	**0.012**
Sex		0.823 (0.155–3.189)	0.630	0.921 (0.193–3.088)	0.696
Clinical stage*	Grade I/II V.S. Grade III/IV	3.862 (0.587–16.542)	0.082	3.121 (0.528–8.664)	0.088
	Grade I V.S. Grade II	1.362 (0.525–3.334)	0.326	1.153 (0.378–2.935)	0.255
	Grade II V.S. Grade III	3.354 (0.815–12.981)	0.058	3.211 (0.664–6.862)	0.120
	Grade III V.S. Grade IV	4.996 (1.878–14.223)	0.069	3.158 (1.232–7.735)	0.076

Abbreviations: CI, confidence interval; HR, hazard ratio.

Boldface highlights statistical significant differences.

### Rspo1 is upregulated in glioma tumor cells upon treatment with radiation

**Figure 3:**
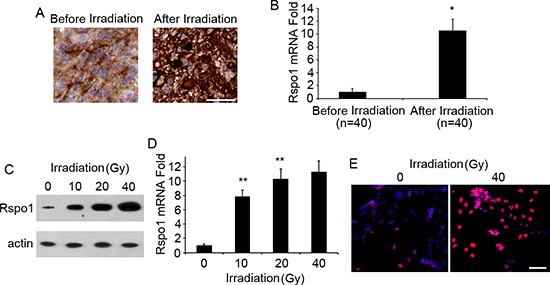
Expression of Rspo1 is increased in glioma tumors and the glioma cell line U87 upon radiation treatment **(A)** Representative image of immunohistochemical staining of glioma biopsies obtained before and after conventional treatment. After chemotherapy, high-intensity cytoplasmic staining indicated higher levels of Rspo1 protein compared to staining obtained before treatment. Scale bar: 50 μm. **(B)** Rspo1 expression was confirmed by qRT-PCR in glioma samples before and after radiation treatment. **(C)** U87 cells were treated with 10, 20 and 40 Gy radiation for 48 h, and Rspo1 protein levels were evaluated by Western blotting; actin was used as a loading control. **(D)** Levels of Rspo1 mRNA in U87 cells treated with 10, 20 and 40 Gy radiation for 48 h as determined by qRT-PCR analysis. **(E)** Upregulation of Rspo1 (in red) was confirmed by immunofluorescence staining following treatment with 40 Gy radiation for 48 h. Nuclei were counterstained with DAPI (in blue). Scale bar: 50 μm. Error bars: ± S.D. *p < 0.05. **p < 0.01. The results represent at least three separate experiments.

To investigate whether radiation treatment can trigger tumor-specific increases in Rspo1 levels in patients with gliomas, we identified patients for whom tumor samples were available both before and after radiation treatment. As shown in Fig. 3A, Rspo1 protein was significantly upregulated after radiation therapy as determined by immunohistochemical analysis. Rspo1 upregulation was also confirmed at the mRNA level by real-time qRT-PCR analysis (Fig. 3B). To further investigate the role of Rspo1 in radiation-treated glioma cells, we selected the glioma cell line U87 as our study model. Both Rspo1 protein and mRNA levels were increased upon exposure to radiation in U87 cells, and this upregulation was concentration-dependent (Fig. 3C, 3D). The upregulation of Rspo1 protein was also confirmed using immunofluorescence staining (Fig. 3E).

### Silencing of Rspo1 potentiates radiation-induced cell death and tumor growth inhibition

**Figure 4:**
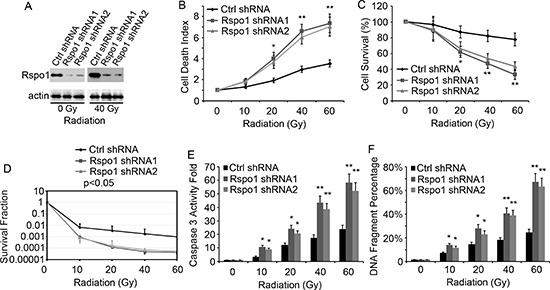
Silencing of Rspo1 sensitizes U87 cells to radiation **(A)** U87 cells transfected with either nonsense control shRNA (Ctrl shRNA) or Rspo1 shRNA1 and Rspo1 shRNA2. The levels of Rspo1 were detected by immunostaining with the anti-Rspo1 antibody, and actin was used as a loading control. **(B, C)** Silencing of Rspo1 sensitizes tumor cells to radiation. U87 cells were transfected with either a nonsense control shRNA (Ctrl shRNA) or one of two specific shRNAs targeting Rspo1. At 24 h after transfection, the cells were treated with increasing doses of radiation. The cell death rate (B), measured using the ToxiLight kit as described in the Materials and Methods section, and survival (C) were evaluated 48 h after treatment. The results were normalized against those obtained with control (untreated) cells. Whereas control shRNA-transfected cells showed a general resistance to radiation treatment, Silencing of Rspo1 strongly induced cell death and decreased cell survival in the presence of radiation. **(D)** Survival fraction curves of U87/control shRNA cells and U87/Rspo1 shRNA cells tested by clonogenic survival assay. **(E, F)** Silencing of Rspo1 triggered apoptosis in combination with radiation treatment. U87 cells were transfected as in B and C and treated with the indicated dose of radiation for 24 h. Active caspase 3 levels (E), normalized to those in untreated cells, and DNA fragmentation (F) were evaluated as described in the Materials and Methods section. Whereas radiation failed to induce apoptosis in control shRNA-transfected cells, Rspo1-silenced cells showed a strong increase in the apoptotic rate. The results represent at least three separate experiments. Error bars: ± S.D. *p < 0.05. **p < 0.01.

The upregulation of Rspo1 upon radiation treatment suggests that the dependence on Rspo1 for survival is amplified in radiotherapy-treated cancer cells. Thus, we analyzed the effect of silencing Rspo1 by shRNA on radiation-induced cell death. U87 cells were transfected with plasmids expressing Rspo1 shRNAs and treated with increasing doses of radiation. Both Rspo1 shRNA1 and Rspo1 shRNA2 silenced Rspo1 protein expression by over 80% compared with nonsense control shRNA (Fig. 4A). Silencing of Rspo1 was associated with a marked potentiation of radiation-induced cell death, as shown by the loss of cell permeability (Fig. 4B) and decrease in cell survival (Fig. 4C). Furthermore, a clonogenic survival assay was performed to measure radiation sensitivity. We found that the survival fraction was significantly decreased when Rspo1 was knocked down by Rspo1 shRNA in U87 cells (Fig. 4D). Activation of the cell apoptosis marker caspase 3 (Fig. 4E) and DNA fragmentation (Fig. 4F) were increased accordingly.

To test whether Rspo1 shRNA is toxic to normal brain cells, we transfected Rspo1 shRNA into normal rat astrocyte D1 TNC1 cells, which express very low levels of Rspo1 (data not shown). We found that Rspo1 shRNA had no effect on D1 TNC1 cell proliferation, as measured by MTT assay (Supplemental Fig. S1A), and had no effect on the radiation sensitivity of D1 TNC1 cells, as measured by a clonogenic survival assay (Supplemental Fig. S1B).

**Figure 5:**
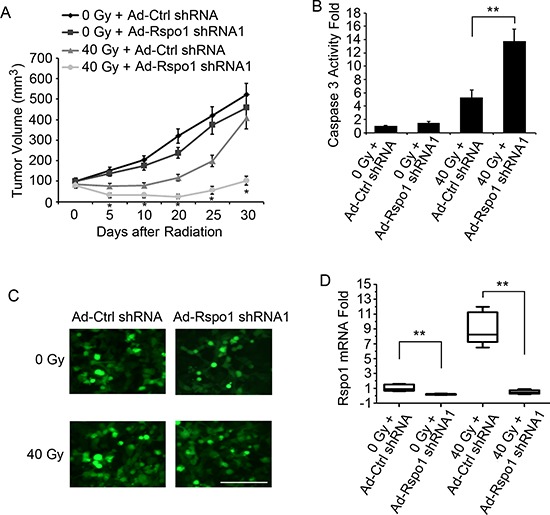
Inhibition of tumor growth by combining Rspo1 silencing and radiation **(A)** Silencing of Rspo1 potentiated the anti-cancer effect of radiation in a preclinical animal model. U87 cells were engrafted into 6-week-old male athymic nude mice. Once tumors reached a volume of 100 mm^3^, the mice were injected intraperitoneally with adenovirus particles expressing control shRNA (Ad-Ctrl RNA) or Rspo1 shRNA1 (Ad-Rspo1 shRNA1) or treated with a combination of Ad-Rspo1 shRNA1 and 10 Gy radiation (tumor area) twice a week for two weeks (40 Gy in total). The histogram represents the growth in tumor volume for each group as a function of time after radiation treatment in days. Whereas radiation or Ad-Rspo1 shRNA alone did not reduce tumor growth, the combination of Ad-Rspo1 shRNA1 and radiation treatment significantly reduced tumor growth. **(B)** Quantification of apoptosis using the caspase 3 activity assay in xenograft lysates analyzed after 2 days of treatment. **(C)** Representative image of the adenovirus infection rate in tumors. **(D)** Expression of Rspo1 was detected in xenograft tumor lysates 48 h after infection with Ad-Rspo1 shRNA1. Error bars: ± S.D. *p < 0.05. **p < 0.01. For A and B, *n* = 10 mice/group. For C, scale bar: 50 μm. The results represent at least three separate experiments.

We then assessed whether the *in vitro* effects described above could be reproduced *in vivo* in a therapeutic setting. Adenovirus particles encoding Rspo1 shRNA1 (Ad-Rspo1 shRNA1) and nonsense control shRNA (Ad-Ctrl shRNA) were produced. U87 cells were engrafted into nude mice, and animals with established palpable tumors were treated once a week by i.p. injection of Ad-Ctrl shRNA1 or Ad-Rspo1 shRNA1 at 1 × 10^8^ pfu/mouse. These injections were administered alone or in combination with 40 Gy of radiation. Single-agent treatment (Ad-Rspo1 shRNA1 or radiation) according to this administration protocol was associated with detectable but weak tumor growth inhibition, which was resolved during the treatment (Fig. 5A). However, co-treatment of radiation and Ad-Rspo1 shRNA1 was associated with stronger and prolonged tumor growth inhibition (Fig. 5A). The stronger and prolonged effect was associated with increased tumor apoptosis. We assessed the apoptosis level in xenografted tumors after 48 h of treatment with radiation alone, Ad-Rspo1 shRNA1 alone or a combination of both agents. Similar results were observed when using U343 cells (Supplemental Fig. S2). As shown in Fig. 5B, radiation alone or Ad-Rspo1 shRNA1 alone failed to significantly induce caspase 3 activity in the tumors; however, the combined treatment triggered significant caspase 3 activation. The adenovirus infection rate after 48 h in tumor tissue was determined (Fig. 5C), and the level of Rspo1 mRNA in the tumor was determined 48 h after adenovirus infection (Fig. 5D).

We next tested whether overexpression of Rspo1 further increased radioresistance. We transfected the Rspo1 plasmid into U87 cells and established stable cell lines. Using a clonogenic survival assay, we found that overexpression of Rspo1 further increased the radioresistance of these cells (Supplemental Fig. S3A and S3B).

## DISCUSSION

Although radiation therapy is the primary adjuvant treatment modality known to increase the survival of patients with malignant gliomas, it is not a cure, and 90% of tumors recur inside the irradiated tumor volume [10, 11]. In this study, we showed that Rspo1 is frequently upregulated in gliomas (43.4%, 102/235), particularly in high-grade glioma (stages III and IV, 52.3%, 68/130), and that a positive association exists between Rspo1 expression and advanced clinicopathological features. In addition, Rspo1 immunoreactivity was inversely correlated with overall survival and disease-free survival in patients diagnosed with glioma, and it was associated with reduced survival after RT-TMZ therapy, further underscoring the clinical significance of Rspo1 in the pathogenesis, prognosis and treatment of glioma. In particular, we showed that radiation treatment triggered a significant increase in the Rspo1 level in patients with gliomas. We showed that this upregulation is associated with increased induction of cell death upon silencing of Rspo1 by shRNA. As a result, we showed that the combination of radiation with silencing of Rspo1 potentiated the inhibition of tumor growth in a xenograft nude mouse model. Our results thus underscore an important role for Rspo1 in the radioresistance of malignant gliomas and provide novel evidence of the potential therapeutic utility of Rspo1 for the treatment of malignant gliomas. Specifically, there is no or very low Rspo1 expression in normal brain cells, which indicates that inhibition may have no effect on normal brain cells. When we inhibited Rspo1 using Rspo1 shRNA in normal rat astrocyte D1 TNC1 cells, we found that Rspo1 inhibition did not have any effect on normal rat astrocyte D1 TNC1 cells. These results indicated that Rspo1 inhibition does not have toxic effects on normal brain cells. However, whether neural stem cells express Rspo1 and the role of Rspo1 in neural stem cells need further study.

Rspo1 was originally identified as a growth factor for intestinal crypt cells in a transgenic mouse model [4]. Systemic administration of Rspo1 decreased the histological and clinical manifestations of dextran sulfate sodium-induced colitis [12] as well as chemotherapy- and radiation-induced oral mucositis in mice [5]. Furthermore, the blood level of Rspo1 was increased after exposure of the mice to whole-body irradiation, and treatment with adenovirally expressed Rspo1 or pure recombinant Rspo1 protein increased the survival rate of lethally irradiated mice [6, 7]. All of these previous studies suggest that Rspo1 plays an important role in radioresistance, allowing tissue repair or regeneration. However, the role of Rspo1 in tumors is still unclear. For the first time, our studies have shown that Rspo1 expression is correlated with the radioresistance of advanced gliomas, which suggests that Rspo1 protects not only normal tissue but also tumor cells from radiation injury. Zhou et al. suggested using a higher dose of chemoradiotherapy to destroy colon tumor cells more effectively based on the chemoradioprotective effects of Rspo1/Slit2 protein in normal tissue [7] Our results thus indicate that treatment with Rspo1 may also enhance tumor cell radioresistance, thereby interfering with the therapeutic effect. It would be interesting to determine whether a tumor therapy dosage window exists between normal tissue radioprotection and tumor cell radioresistance induced by Rspo1. Additionally, it would be very interesting to test the radiation dose for tumor therapy. Combined treatments involving tumor-specific silencing of Rspo1 combined with non-tumor-specific Rspo1 treatment would be useful, as such treatments could both decrease tumor cell radioresistance and increase normal cell radioprotection.

R-Spondins are a family of secreted proteins that are potent activators of the Wnt-β-catenin pathway. Rspo1 has been demonstrated to bind with high affinity to the Wnt co-receptor, LRP6, to induce phosphorylation, stabilization and nuclear translocation of cytosolic β-catenin, thereby activating TCF/β-catenin-dependent transcriptional responses in intestinal crypt cells [13, 14]. The mechanism by which Rspo1 regulates glioma cell radioresistance requires further investigation. Recently, Li et al. reported that Rspo1 is expressed by neurons in the ventromedial nucleus of the hypothalamus (VMH), and injection of Rspo1 into the third cerebral ventricle inhibited food intake [15]. The question of whether Rspo1 can protect normal brain tissue from radiation injury requires further investigation.

In general, glioblastomas are considered radioresistant tumors, as different radiation modalities have failed to control them in the clinic. Multiple mechanisms have been proposed to be associated with radioresistance in human glioblastoma cells. Recently, cancer cells with stem cell-like properties have been described in a wide range of human tumors. The cancer stem cell model suggests a hierarchical organization of tumors in which a subpopulation of tumor cells at the apex drives and maintains human tumors [16]. Glioma stem cells were also identified and were reported to be more resistant to radiation compared with matched non-stem glioma cells [17–19]. It would be very interesting to test whether glioma stem cells express Rspo1 and whether Rspo1 inhibition increases the radiation sensitivity of glioma stem cells.

Tumors are complex and diverse, and almost every tumor is unique, which is why finding an effective cure for all tumors is challenging. To increase our understanding of tumors, similar tumors can be analyzed to determine their common features. In the present study, we demonstrated that a subpopulation of glioma cells express Rspo1, and this subpopulation of gliomas is radioresistant. Silencing of Rspo1 in this subpopulation of gliomas could produce good therapeutic effects when combined with radiation therapy.

## MATERIALS AND METHODS

### Ethics statement

Informed consent was obtained from all patients enrolled in this study, and the study protocol was approved by the Clinical Research Ethics Committee of Guangdong Medical College. The protocols for all animal studies were also approved by the Clinical Research Ethics Committee of Guangdong Medical College.

### Immunohistochemical staining

Human glioma samples were obtained from the Affiliated Hospital of Guangdong Medical College. The pathological grade of these tumors was defined according to the 2007 WHO criteria. Normal brain samples were obtained from automobile accident victims without glioma. Immunohistochemical staining was performed as previously described.^17^ Tissue sections (5 μm thickness) were deparaffinized, and endogenous peroxidase was quenched using 3% H_2_O_2_ in methanol for 30 min. The sections were incubated in a solution of 10% BSA in PBS at 37°C for 1 h to block non-specific binding; then, they were incubated with IgG (control) or specific antibodies in PBS containing 10% BSA at 4°C overnight. Subsequently, the sections were incubated with a horseradish peroxidase anti-rabbit antibody. Immunoreactivity was detected using the ImmunoPure Metal-Enhanced Diaminobenzidine (DAB) Substrate Kit (Pierce), and the tissue sections were counterstained with hematoxylin. For determination of Rspo1 immunoreactivity, cytosolic or nuclear staining of yellowish or brownish granules was graded using the following scale: 0 for background staining, 1 for faint staining, 2 for moderate staining and 3 for strong staining. The intensity of the immunoreactivity signal was determined relative to that of U87 cells transfected with the Rspo1 plasmid, which also served as a positive staining control. The staining intensity of the control was arbitrarily designated as 3. In addition, the number of immunopositive cells expressed as a percentage of the total cell number throughout the entire tissue section was graded according to the following scale: 0 for < 5%, 1 for 5–25%, 2 for 26–50%, 3 for 50–75% and 4 for 75%–100%. The immunostaining intensity and the percentage of positive tumor cells were multiplied to produce a weighted score for each tumor specimen. Weighted scores of 0–2 and ≥ 3 were defined as negative and positive staining, respectively. [20]

### Plasmids, antibodies and chemicals

Short hairpin RNA (shRNA) directed against Rspo1 was purchased from Thermo (U.S.). The Rspo1 shRNA1 target sequence was 5^′^-TACACTTGGTGCAGAAGTT-3^′^, the Rspo1 shRNA2 target sequence was 5^′^-TGCACTTGTTCATGTCGGG-3^′^, and the nonsense shRNA sequence was 5^′^-TACGCATCCGCAACTGCAG-3^′^. The rabbit anti-Rspo1 antibodies used for Western blotting, immunoprecipitation and immunohistochemical assays were purchased from Abcam. The mouse anti-β-actin antibodies used for the immunoblotting assay were purchased from Sigma. The secondary antibodies anti-mouse IgG-HRP and anti-rabbit IgG-HRP were purchased from Sigma.

### Cell culture

The human glioma cell lines U138, H-4, HS-683, U-87, U-251, and U-343 were purchased from the American Type Culture Collection (ATCC). The cells were cultured according to the recommendations from the ATCC. U87 cells were transfected with plasmids containing Rspo1 shRNA or Ctrl shRNA using Lipofectamine 2000 (Invitrogen). Positive transfectants were selected by incubating cells with 0.8 mg/ml G418 (GIBCO BRL) for two weeks to obtain a stable cell line for Rspo1 silencing in subsequent assays.

### qRT-PCR

qRT-PCR was performed as described.^17^ Total RNA was extracted using the Absolutely RNA Miniprep Kit (Stratagene) and reverse transcribed using the ThermoScript RT-PCR System (Invitrogen). The cDNA was used in real-time PCR with SYBR-Green Master PCR Mix (Applied Biosystems), and triplicate reactions were performed. All RT^2^ and qPCR primer pairs were purchased from SABiosciences. PCR and data collection were performed using the Step-one qPCR System (Stratagene). All expression levels were normalized to the expression levels of endogenous β-actin, which served as a control. The relative expression level for each target gene was compared with that of the calibrator gene (β-actin) and expressed as 2-(Ct-Cc) (Ct and Cc are the mean threshold cycle differences after normalization to β-actin). The relative expression levels of the samples are presented using a semi-log plot.

### Immunoblotting

For immunoblotting, the samples were subjected to SDS-PAGE, transferred to PVDF membranes (Millipore) and detected using the appropriate primary antibodies followed by horseradish peroxidase-conjugated goat anti-mouse or anti-rabbit IgG. The blotting signals were detected using SuperSignal West Dura Extended Duration Substrate (Pierce). Quantitative analysis of the immunoblotting signals was performed by densitometry using LAS4000 Image Software (Fuji Film).

### Immunofluorescence staining

Cells were fixed with 4% paraformaldehyde and permeabilized with 0.05% Triton X-100 in PBS at room temperature for 20 min. The samples were blocked with 1% bovine serum albumin (BSA; Sigma) and incubated with the appropriate primary antibody at 37°C for 1 h. After extensive washing, the samples were incubated with Alexa Fluor 488-labeled goat anti-rabbit IgG at 37°C for 1 h. The cells were then washed and mounted for observation under a scanning confocal microscope (TCS SP2 Leica).

### Cell death assay and conventional radiation treatment

Exponentially growing U87/v cells, U87/Rspo1 shRNA cells and U87/Rspo1 shRNA2 cells were trypsinized and irradiated at 10, 20, 40, and 60 Gy. The radiation was derived from a 4 MV Linear Accelerator (Varian, USA), and the dose rate was 320 mu/min. After irradiation, the cells were counted. For total cell death assays, 5 × 10^3^ cells per well were grown in 96-well plates in serum-poor medium and treated with radiation. After 48 h, cell death was evaluated using the bioluminescence cytotoxicity assay ToxiLight (Lonza, Basel, Switzerland) according to the manufacturer's instructions.

Cell survival was measured using the MTS assay according to the manufacturer's instructions (CellTiter 96 AQueous One Solution Cell Proliferation Assay, Promega). Cells (3 × 10^3^) were grown in a 96-well plate in serum-poor medium for 16 h, treated with the indicated radiation dose in serum-free medium, and cultured for 48 h.

Caspase 3 activity was assayed using the caspase3/CPP32 Fluorimetric Assay Kit (Gentaur Biovision, Brussels, Belgium) according to the manufacturer's instructions. Caspase 3 activity (activity/min/μg of protein) was calculated from a 1 h kinetic cycle reading on a spectrophotometer (405 nm/510 nm, Infinite F500, Tecan, Männedorf, Switzerland). DNA fragmentation was measured by immunofluorescence staining with an antibody against rH2X, and the staining sensitivity was determined.

### Clonogenic assay

Exponentially growing U87/v cells, U87/Rspo1 shRNA cells and U87/Rspo1 shRNA2 cells were trypsinized and irradiated at 10, 20, 40, and 60 Gy. The radiation was derived from a 4 MV Linear Accelerator (Varian, USA), and the dose rate was 320 mu/min. After irradiation, the cells were counted to determine the final cell number required for each colony-forming experiment in 6-well plates. For example, to obtain 150–250 colonies per well in control wells after the incubation period, 1000 cells were plated. Each radiation dose and untreated control was set up in triplicate wells. All plates were incubated at 37°C for 8–14 days depending on the cell growth rate. The plates were then fixed with 70% ethanol, and the colonies were stained with 1% Giemsa solution. Colonies that consisted of more than 50 cells were scored. At least three independent experiments were performed for each cell line. The cell survival curves were drawn by plotting the means of three experiments.

### Generation of adenovirus expressing Rspo1 shRNA

Custom-made high-titer adenoviruses expressing nonsense control shRNA and Rspo1 shRNA1 (sequence: 5′-TACACTTGGTGCAGAAGTT-3′) were purchased from Vector Biolabs (U. S., PA), and 1 × 10^8^ pfu adenoviral particles containing the above-mentioned constructs were injected into the tail vein of nude mice bearing tumors. After 48 h, the tumors were collected for determination of Rspo1 expression by immunofluorescence staining or by qRT-PCR.

### Animal model

Six-week-old (18–20 g body weight) male athymic nu/nu mice were obtained from SLAC Laboratory Animals (China, Shanghai). The mice were housed in sterilized filter-topped cages and maintained in a pathogen-free animal facility. U87 cells were implanted by s.c. injection of 107 cells in 100 μl of PBS into the right flank of the mice. Once the tumors were established (V≈100 mm^3^), the mice were treated with adenovirus expressing Rspo1 shRNA and/or radiation for 2 weeks. For the radiation treatment, the mice were anesthetized using ketamine (100 mg/kg, i.p.), and the tumor area was irradiated at 40 Gy. The radiation was derived from a 4 MV Linear Accelerator (Varian, USA), and the dose rate was 320 mu/min. Tumor sizes were measured with a caliper. The tumor volume was calculated with the formula *V* = [*L* × *W*^2^] × 0.52 (*V* = volume, *L* = length, and *W* = width). Ten animals from each group were sacrificed 48 h after the initial treatment to evaluate caspase 3 activation in tumor tissue and to quantify Rspo1 expression in engrafted tumor cells. The data were analyzed using Student's *t*-test, and *p* < 0.05 was considered statistically significant.

### Statistical analysis

Overall survival is represented in months and defined as the interval between the date of the surgery and the date of death or the last follow-up. Overall survival curves were estimated using the Kaplan–Meier method, and the difference in survival was evaluated using the log-rank test. *p* < 0.05 and 0.01 were defined as statistically significant and very significant, respectively. All computations were performed using R 2.9.0 software (http://www.r-project.org).

## SUPPLEMENTARY MATERIALS, METHODS, FIGURES AND TABLES


