# Supplementary material for: Silencing of R-Spondin1 increases radiosensitivity of glioma cells
Source: Oncotarget. 2015 Mar 12;6(12):9756–65. doi: 10.18632/oncotarget.3395 (PMC4496395; doi:10.18632/oncotarget.3395)
Supplement: Supplementary file 1 [file oncotarget-06-9756-s001.pdf]

SUPPLEMENTARY MATERIALS AND METHODS

Cell culture

The normal rat astrocyte D1 TNC1 Cells were purchased from the American Type Culture Collection (ATCC). The cells were cultured according to the recommendations from the ATCC. D1 TNC1 cells were transfected with plasmids containing Rspo1 shRNA or Ctrl shRNA using Lipofectamine 2000 (Invitrogen). Positive transfectants were selected by incubating cells with 0.8 mg/ml G418 (GIBCO BRL) for two weeks to obtain a stable cell line for Rspo1 silencing in subsequent assays.

MTT assay

The D1 TNC1 cells transfected with control shRNA or Rspo1 shRNAs, were grown to the exponential phase and detached through trypsin treatment. A total of 2500 cells/ml were plated onto 96-well tissue culture plates (100 μl complete medium/well) and cultured at 37°C in 5% CO2. At different time points, 10 μl per well MTT reagent was added and incubated at 37°C for 4 h. Subsequently, 100 μl DMSO was used to terminate the reaction, and the optical density was determined at OD57/OD630 nm using a multi-well plate reader. The data from three independent experiments were analyzed using Student's *t*-test, and $p < 0.05$ was considered statistically significant. 2

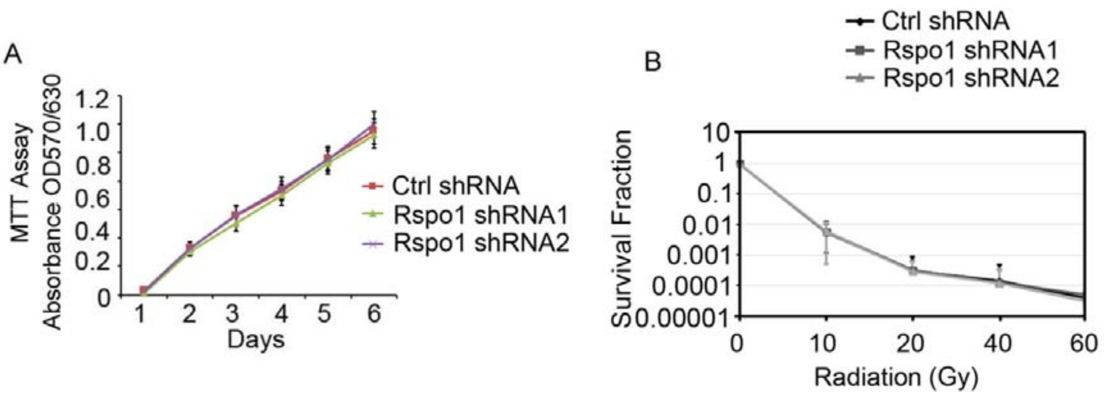

Supplementary Figure S1: Silencing of Rspo1 has no effect on normal rat astrocyte D1 TNC1 Cells. (A) D1 TNC1 cells transfected with either nonsense control shRNA (Ctrl shRNA) or Rspo1 shRNA1 and Rspo1 shRNA2. The proliferation ability of cells were measured using MTT assay. **(B)** Survival fraction curves of D1 TNC1/control shRNA cells and D1 TNC1/Rspo1 shRNAs cells tested by clonogenic survival assay.

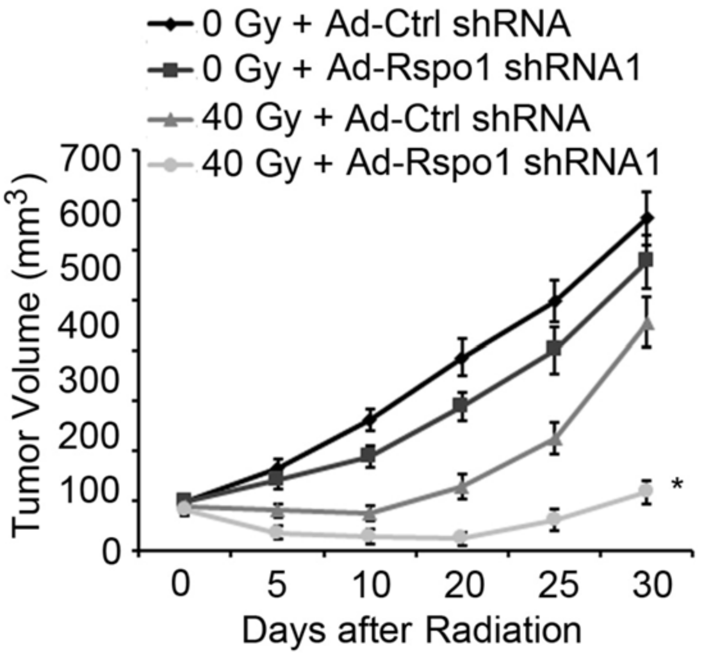

Supplementary Figure S2: Silencing of Rspo1 potentiated the anti-cancer effect of radiation in a preclinical animal model using U343 cells. U343 cells were engrafted into 6-week-old male athymic nude mice. Once tumors reached a volume of 100 mm^3, the mice were injected intraperitoneally with adenovirus particles expressing control shRNA (Ad-Ctrl RNA) or Rspo1 shRNA1 (Ad-Rspo1 shRNA1) or treated with a combination of Ad-Rspo1 shRNA1 and 10 Gy radiation (tumor area) twice a week for two weeks (40 Gy in total). The histogram represents the growth in tumor volume for each group as a function of time after radiation treatment in days. Whereas radiation or Ad-Rspo1 shRNA alone did not reduce tumor growth, the combination of Ad-Rspo1 shRNA1 and radiation treatment significantly reduced tumor growth. *$p < 0.05$.

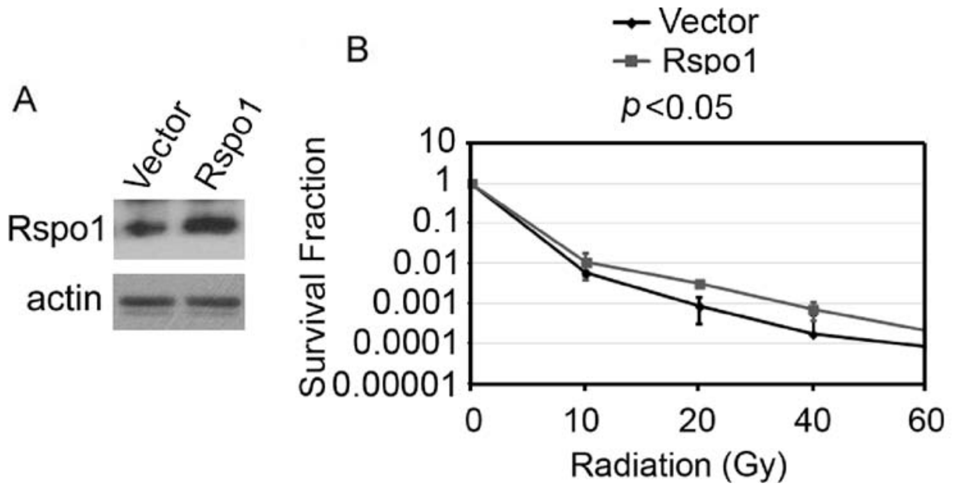

Supplementary Figure S3: Overexpression of Rspo1 further increase the radioresistance. **(A)** U87 were transfected with vector control or Rspo1 plasmid, the expression of Rspo1 were measured using Western Blot assay with anti-Rspo1 antibody, the actin were used as loading control. **(B)** Survival fraction curves of U87/V cells and U87/Rspo1 cells tested by clonogenic survival assay

Supplementary Table S1. 45 patients in whom tumors were available both before and after RT. The time when samples were collected after RT for each patients were indicated in this table

Patients Number	Pathological-grade	Sex	Age	Timing after radiation (Days)
1	II	Male	33	31
2	II	Male	38	52
3	II	Male	42	44
4	II	Male	44	48
5	II	Male	45	66
6	II	Male	56	136
7	II	Male	58	62
8	II	Male	66	48
9	II	Male	56	72
10	II	Male	58	48
11	II	Male	44	35
12	II	Male	45	53
13	II	Male	56	42
14	II	Male	63	33
15	II	Male	56	56
16	II	Male	58	78
17	II	Male	39	125
18	II	Male	55	120
19	III	Male	57	92
20	III	Male	68	68
21	III	Male	72	132
22	III	Male	75	84
23	III	Male	68	88
24	III	Male	57	68
25	III	Male	72	42
26	III	Male	59	50
27	III	Male	63	128
28	III	Male	61	32
29	III	Male	55	35
30	III	Male	57	58
31	III	Male	68	170
32	II	Female	62	162
33	II	Female	65	88
34	II	Female	82	60
35	II	Female	43	50

(*Continued*)

Patients Number	Pathological-grade	Sex	Age	Timing after radiation (Days)
36	II	Female	52	42
37	II	Female	65	35
38	II	Female	58	70
39	II	Female	49	75
40	III	Female	55	48
41	III	Female	64	50
42	III	Female	67	56
43	III	Female	71	60
44	III	Female	68	68
45	III	Female	65	55
